# More Land, Less Pollution? How Land Transfer Affects Fertilizer Application

**DOI:** 10.3390/ijerph182111268

**Published:** 2021-10-27

**Authors:** Junqian Wu, Xin Wen, Xiulin Qi, Shile Fang, Chenxi Xu

**Affiliations:** 1 China Western Economic Research Center, Southwestern University of Finance and Economics, Chengdu 610074, China; wujunqian@swufe.edu.cn (J.W.); wenxin20111122@126.com (X.W.); 2 Business School, Zhengzhou University, Zhengzhou 450001, China; xuchenxizzu@163.com; 3 School of Economics, Zhejiang Gongshang University, Hangzhou 310000, China; fangshile1989@163.com

**Keywords:** fertilizer application intensity, land transfer, land size, mechanization

## Abstract

Reducing fertilizer use is key to curbing agricultural pollution and ensuring food safety. Land transfer enables farmers to obtain a more appropriate production scale, but its effect on the intensity of fertilizer application is not theoretically certain. On one hand, farmers with more land may adopt more scientific production methods, thus reducing the use of chemical fertilizers. On the other hand, the short-term behavior of land grantees on transferred land may increase fertilizer use intensity. This paper attempts to theoretically elucidate the specific mechanisms by which land transfer affects the intensity of fertilizer application and to verify the relationship between the two using data from fixed rural observation sites across China from 2011–2014 with the fixed-effects model and the mediating effect model. This paper concludes that (1) land transfer significantly reduces the intensity of fertilizer use; (2) land transfer increases the land size and promotes the use of machinery by farmers, but only the increase in land size further reduces the intensity of fertilizer application; (3) the effect of land transfer on fertilizer application intensity is significant only for food crops and not for cash crops, and (4) the effect of land transfer on fertilizer application intensity is most pronounced in western China, where land fragmentation is the severest and insignificant in eastern China, where agricultural modernization is more advanced.

## 1. Introduction

Fertilizer is an important material input in agricultural production and plays a vital role in increasing crop yields. The rapid development of the fertilizer industry has ensured an adequate supply of fertilizers, and the use of fertilizers in China has shown a rising trend [1]. However, China’s overuse and inefficient use of chemical fertilizers is a serious problem [2]. From the data, the amount of fertilizer used in China far exceeds the accepted safety limit of 225 kg/hm^2^ in developed countries, and the utilization rate of fertilizer is less than 50% of that in developed countries. This, on the one hand, increases the cost and reduces the technical efficiency of agricultural production. On the other hand, it also brings serious agricultural surface pollution, which in turn leads to arable land slabbing, soil acidification, and eutrophication of water bodies [3,4,5,6,7]. For this reason, in 2015, China proposed that during the 13th Five-Year Plan period, food production will no longer focus on the pursuit of increased production as the only goal but will emphasize the supply of safe, high-quality agricultural products. Achieving a reduction in the use of chemical fertilizers is crucial to the green development of Chinese agriculture and the improvement of the global ecological environment.

In the context of the household contract responsibility system, the small farmer economy is an important reason why Chinese farmers apply more chemical fertilizers than the world average [8]. For a long time, China’s agricultural modernization has been based on the Japanese model, which emphasizes more agrochemical inputs to compensate for the lack of land endowment [3]. However, with the rapid industrialization and urbanization of China, the relative prices of agricultural production factors have changed, especially the labor cost. In this context, China’s agricultural modernization path began to shift to a more labor-saving American model. The development of a moderate-scale economy has thus become an inevitable trend in agricultural development [9]. Along with the steady progress of the reform of the “three rights of rural land” system, China’s rural land transfer has gradually accelerated, and the proportion of land transfer has now reached one-third of the total arable land area.

Various impacts of land transfer have been well discussed in previous studies. In particular, there are a considerable number of studies on how land transfer affects farmers’ income and the technical efficiency of agricultural production, and the findings are primarily consistent [10,11,12]. The factors influencing farmers’ fertilizer input behavior have also been discussed extensively in the existing literature, mainly regarding farmers’ own characteristics, land size, agricultural system, resource endowment, and cropping structure [13,14,15,16]. A series of farmers’ own characteristics will directly influence their agricultural production decisions, which are also crucial factors affecting their fertilizer inputs [17,18]. On the one hand, farmers with smaller arable land, long years of farming experience, and male heads of households tend to apply less fertilizer. On the other hand, younger farmers and farmers with higher awareness of low-carbon agriculture or membership in professional farmer cooperatives tend to apply less fertilizer [19].

Most studies on land size have shown that as land size increases, fertilizer application intensity decreases with no loss or even increase in yield [20]. However, some empirical studies have also shown that fertilizer use efficiency is inversely proportional to farm size; i.e., the smaller the farm size, the higher the fertilizer use efficiency [21]. The property right system of agricultural land is also an essential factor affecting the fertilizer application behavior of farmers. Such influences include the following: first, unclear property rights of farmland lead to the absence of environmental management institutions, which cannot effectively regulate and supervise agricultural surface source pollution behaviors; second, unstable contract management rights of farmland induce short-term behaviors of farmers; third, fragmentation of land makes it impossible to implement uniform environmental behaviors; finally, an imperfect farmland transfer system makes farmers lose their incentive to invest in land, leading to rough land management [22]. Some studies have found both direct and indirect effects of agricultural land endowment on fertilizer input intensity. The direct effect is that with a low level of agricultural land endowment, the input of production factors such as fertilizer has to be increased to increase food production. The indirect effect is that higher farmland endowment reduces food imports, increasing fertilizer inputs [3]. The structural adjustment of China’s cropping industry has brought about a decreasing share of the sown area of crops represented by grain in the total sown area of crops, which has also led to an increasing intensity of fertilizer application in agriculture [23]. In addition, with the development of China’s agricultural land transfer market, an increasing number of farmers are cultivating their own land and the land transferred to them [24]. It has been found that farmers are less likely to apply organic fertilizer and use it less on transferred land than on their own land. However, this difference in investment is narrowing as farmers’ expectations of land lease stability increase [25]. Another reason why land transfer may increase the intensity of fertilizer application is that the significant shift in rural labor it brings will reduce agricultural labor. This prompts farmers to use chemical fertilizers to replace organic fertilizers that require more labor [26].

The most relevant to this paper is Wu et al. [27]. They found that fertilizer application per unit area decreases with farm size, and yields are not significantly affected. On one hand, differences in land size lead to different combinations of factor inputs, and large-scale farms are more likely to substitute advanced agricultural technologies for fertilizer use. On the other hand, farmers running large-scale farms tend to have better agricultural knowledge and management skills and can use agricultural input factors more efficiently. In addition, the paper argues that the main reason for China’s failure to achieve land scale expansion through land transfer is the existence of the land system and the hukou system. This implies that if the institutional distortions are removed, the intensity of fertilizer application in China will decrease with land size expansion. However, there are some shortcomings in the research content and empirical methods of this paper. First, although the paper proposes two theoretical mechanisms underlying the influence of land size on fertilizer application intensity, it does not validate them through empirical models. Second, the domestic microdata used in the empirical test of this paper are cross-sectional data, which cannot solve the problem of omitted variables caused by the heterogeneity that does not change over time, thus reducing the robustness of the conclusions.

In general, previous literature has systematically studied the impact of the land transfer on agricultural development and farmers’ income. However, there are fewer studies on the effect of land transfer on fertilizer input behavior. Further, it is not theoretically certain whether the land transfer will reduce the intensity of fertilizer use or not. In terms of disincentives, the increase in land size and mechanization will reduce the intensity of fertilizer application. In turn, from the perspective of facilitation, the short-term behavior of land recipients increases the intensity of fertilizer application. The lack of theoretical clarity is also reflected in the conflicting results of relevant empirical studies. Therefore, there is a strong need to explore further the mechanisms by which land transfer affects fertilizer application intensity. In addition, most previous empirical studies have used cross-sectional data or data based on a specific region, and their results are subject to more significant interference from omitted variables and sample selection problems [2,12,21,27]. In view of the limitations of previous studies, this paper analyzes in detail the possible mechanisms by which land transfer affects fertilizer application intensity not only theoretically but also empirically, using micro panel data from 2011–2014 from fixed observation sites in rural China to overcome the endogeneity and sample selection problems to verify the causal relationship between the two.

## 2. Theoretical Analysis

It is not theoretically certain whether a land transfer will reduce the intensity of fertilizer application or not.

On the one hand, a land transfer can reduce the intensity of fertilizer application by increasing the size of land and prompting farmers to increase mechanization rates. First, according to the theory of induced technological change, it is rational to rely on biochemical technologies such as fertilizers to increase land yield in farming countries with large populations and small landholdings [28,29]. This also implies that if the land size increases, there may be substitution of fertilizer application by other technologies. Second, as rural laborers continue to move to cities and non-farm industries, land transfer is becoming more common in rural China, providing conditions for expanding agricultural operations. According to the theory of scale economy, expanding the scale of operation can reduce the average cost. Small farmers expand their operation scale through agricultural land transfer to reach or gradually approach the optimal production scale of farmers, thus improving the technical efficiency of farmers’ agricultural production. Therefore, the technical efficiency of land transferees may be higher than that of other farmers [7,8]. At a higher level of technology, farmers seeking to maximize their profits have more room to adjust their original factor input methods. Behaviors such as excessive input of chemical fertilizers, which are harmful to long-term development, will be adjusted. Last, the expansion of land size will lead to a shift in the production pattern of farmers from the original diversification to monoculture. This shift will further enhance farmers’ experience in production and thus optimize their fertilizer application behavior. In addition, with the expansion of production scale comes the expansion of operation scale. When a farmer’s production scale is small, it is not rational to pursue the quality of agricultural products because of his/her weak bargaining power in the market. However, when the farmer has a certain production scale, the increased bargaining power will motivate him/her to pursue markets with higher profitability. This leads to changes in production behavior, such as a shift from conventional crops to organic crops, and will further reduce the intensity of chemical fertilizer application.

The scale of agricultural land operation must match the supply of production factors, such as capital, technology, and labor, to achieve the optimal allocation of factors [30]. The application of advanced science and management techniques is enough to improve the efficiency of using material input factors such as fertilizers. The application of these technologies has higher fixed costs. However, this would be diluted by a larger land scale [10]. When the production scale reaches a certain level, sizeable agricultural machinery provides the technical conditions for a change in how fertilizer is applied in agriculture, allowing a shift from traditional fertilizer application methods to more sophisticated fertilizer application methods with smaller amounts [31]. More specifically, mechanical operations require a larger land size, and overly large plots of land can reduce the cost of mechanical operations. When land transfer brings about an increase in land size, mechanization will reduce the intensity of fertilizer application. This is because mechanization can avoid the irregularities of manual fertilizer application. In addition, more systematic fertilizer application can increase the traceability of fertilizer dosage, thus curbing the intensity of fertilizer application.

On the other hand, farmers may apply more fertilizer on the transferred land due to moral hazards. Especially when the transfer land’s contract duration and stability are not clear, farmers are more likely to develop short-term behavior and thus apply more fertilizer on the transferred land [32]. According to property rights theory, stable, clear, and long-term land rights will encourage farmers to make investments that can lead to soil improvement in the long term, such as the application of organic fertilizer [33,34,35]. Conversely, unstable land rights can lead to predatory short-term production behavior of farmers. Since the duration of the contract period of the land transferee cannot exceed the duration of the land contract of the land transferor, the short-term behavior of farmers’ transfer to the land is actually a rational choice under various constraints. Considering the unstable policy expectations and the general lack of property rights protection in rural China, farmers are actually risk-averse, applying more fertilizer on the transferred land to obtain returns in less time. It has been shown that the higher the expectation of farm households for long-term use of agricultural land, the higher their probability of applying organic fertilizer [36]. Moreover, the fertilizer application behavior will vary with the type of land transferred. The probability and amount of organic fertilizer application by farmers on farmland transferred from non-relatives is significantly lower than those on farmland transferred from relatives, which is also a manifestation of the short-term behavior of land grantees [25,36].

In addition, land size may also be a key factor that weakens the impact of the land transfer on the intensity of fertilizer application. Large-scale land transfer can lead to the creation of professional farmers. Compared with traditional farmers, professional farmers care more about long-term returns and are able to make full use of land scale and science and technology in their production. Therefore, they will apply chemical fertilizers less intensively [37]. However, most of the land grantees are still small farmers in China who have very limited land to transfer into. By the end of 2016, the area transferred accounted for 35.1% of the area contracted by families. Among them, the area transferred into farm households accounted for 58.38% of the total area transferred. The production practices of small-scale land transferees are not fundamentally different from those of ordinary farmers. They may even increase the intensity of fertilizer application in pursuit of profit because of moral hazards.

Based on the above analysis, we propose the following hypotheses for this paper.

**Hypothesis 1.**  *Land transfer will reduce the intensity of fertilizer application.*

Alternative **Hypothesis 2.**  *Land transfer will increase or not affect the intensity of fertilizer application.*

## 3. Research Design

### 3.1. Data

The data used in this paper are the 2011–2014 farm household tracking survey data from China’s rural fixed observation sites. The database was first built in 1984 and organized by the Policy Research Office of the Central Committee of the Communist Party of China and the Ministry of Agriculture of China. It has the advantages of high data stability, wide coverage, large sample capacity, and rich survey indicators. The indicators in this database cover various aspects of production, consumption, employment, and living of farm households, providing panoramic production and living data information of Chinese farm households at the micro level. Among them, the variables of farm households’ subcontracting into the arable land area, year-end arable land area, and the quantity of fertilizer used by farm households provide support for the construction of the core variables of this paper. In addition, the rich indicators of control variables also equip us with the ability to identify the causal relationship between land transfer and fertilizer use. After dealing with outliers and missing value issues, there are more than 12,000 farm households in the sample used for the analysis of this paper. The sample is unbalanced panel data.

### 3.2. Variables

#### 3.2.1. Dependent Variable

The dependent variable in this paper is the intensity of fertilizer application (IFA). The IFA indicator is measured by the average fertilizer application per mu of farm households in the survey questionnaire.

#### 3.2.2. Independent Variable

The core explanatory variable in this paper is the ratio of transferred land (RTL). To measure the level of land transfer to grantees, there are generally three indicators: (1) the ratio of transferred land, (2) the presence or absence of land transfer, and (3) the total area of transferred land. Of these three indicators, the presence or absence of land transfers is too coarse a measure, and much of the heterogeneous information in the data is lost because land endowment varies greatly among regions in China, and the area of land contracted by farmers varies by household size. Therefore, the same area of transferred land may mean very different levels of land transfer for different households. Therefore, this paper mainly uses the ratio of transferred land as an indicator to measure the level of land transfer. Specifically, the land transfer ratio is measured as the ratio of the farmer’s transferred land area to the year-end operating area.

We quadrupled the sample according to the level of land transfer, and Level 1 represents the lowest transfer level, while Level 4 represents the highest transfer level. The distribution of the sample at different transfer levels is shown in Table 1.

#### 3.2.3. Control Variables

Following the previous studies, this study controls for individual characteristics of household heads with the household head’s age, the household head’s education level, and whether the household head received agricultural training. The household characteristics are controlled by the household income, the proportion of total agricultural labor time of the household, and the total land operation scale of the household. Among them, the proportion of total agricultural work time of farm households reflects the degree of labor transfer of farm households. The definitions and measures of the control variables are shown in Table 2.

Again, the sample is quadratically divided according to the level of land transfer, and the average value of the intensity of fertilizer application and the control variables are presented in Table 3. As shown in Table 3, the average age of household heads is younger and more educated as the level of land transfer increases. Moreover, the proportion of agricultural training, the average number of plots, and the percentage of agricultural labor hours are higher for farmers who transferred to more land. More critically, the intensity of fertilizer application by farm households decreases significantly with the level of land transferred. This initially verifies H1.

### 3.3. Model

#### 3.3.1. Fixed Effects Model

Because the sample used in this paper is panel data, and the *p*-value of the Hausman test is less than 0.05, a fixed-effects model is established [38]. The regression equation is as follows:

(1) $$IFA_{it}=\theta +\alpha RTL_{it}+\beta X_{it}+\mu_{i}+\lambda_{t}+\varepsilon_{it}$$

The dependent variable $IFA_{it}$ is the intensity of fertilizer application. The key independent variable $RTL_{it}$ measures the level of land transfer. $X_{it}$ consists of a set of control variables capturing farmers’ characteristics discussed in detail in Section 3.2.3. $u_{i}$ and $\lambda_{i}$ represent the city fixed effect and time effect, respectively.$\varepsilon_{it}$ represents the error term. α is used to measure the effect of land transfer on the intensity of fertilizer application.

#### 3.3.2. Mediating Effect Model

To examine the mechanism by which land transfer affects the intensity of fertilizer application, we set up the following mediating effect model with reference to Baron and Kenny [39].

(2) $$IFA=\theta_{1}+\alpha_{1}RTL+\beta_{1}X+\varepsilon_{1}$$

(3) $$\mathrm{intermediate}=\theta_{1}+\alpha_{2}RTL+\beta_{2}X+\varepsilon_{2}$$

(4) $$IFA=\theta_{3}+\alpha_{3}RTL+\lambda_{3}\mathrm{intermediate}+\beta_{3}X+\varepsilon_{3}$$

Intermediate is the mediating variable. Based on the discussion in the Theoretical Analysis section, this paper focuses on the role played by the farmer’s land size and mechanization level as mediating variables in the relationship between land transfer and fertilizer application intensity.

## 4. Results and Discussion

### 4.1. Baseline Regression

We first performed a baseline regression on the relationship between land turnover level and fertilizer application intensity. The regression results reported in Column (1) of Table 4 indicate that the level of land transfer can significantly reduce the intensity of fertilizer application by farm households. This result verifies H1. To ensure the robustness of the conclusions, we constructed two new indicators, RTL1 and RTL2, to measure the level of land turnover and regressed them still using fertilizer application intensity. Specifically, RTL1 is measured by whether the farmer transferred to the land or not (yes = 1; no = 0), and RTL2 is measured by the total area of land transferred by the farmer. The regression results are shown in Columns (2) and (3) of Table 4, indicating that the negative relationship between the level of land transfer and fertilizer application intensity is robust. These results validate H1.

According to previous studies, there are negative and positive mechanisms for the effect of land transfer on fertilizer application intensity. Between them, the negative effect of land transfer on fertilizer application intensity mainly comes from the adjustment of farmers in terms of production technology [29]. The larger scale of land not only gives farmers more room to substitute fertilizer application with other technologies but also provides them with positive incentives to make such adjustments [7,8,10,31]. In contrast, the positive effect of land transfer on the intensity of fertilizer application comes mainly from the opportunistic behavior of farmers. Farmers are more inclined to gain short-term gains by increasing fertilizer application intensity on transferred land compared to their own land [32,36]. Our findings suggest that land transfer reduces fertilizer application intensity in general. The reason for this is that not only has land transfer in rural China been prohibited for a long time in the past, but the land owned by each farming household is minimal under the family contract system [8]. Under this constraint, according to the theory of induced technological change, using more fertilizers to enhance mu yield is rational for farmers [28,29]. This implies that the amount of fertilizer applied in rural China was high before the beginning of land transfer [2]. Moreover, when the land transfer started, other technologies began to replace the role of chemical fertilizers in agricultural production, and the amount of chemical fertilizer application gradually decreased. In addition, in rural China, the opportunistic behavior of farmers in transferring land may be lower than expected. This is first because rural China is a “society of human relations”, and morality and public opinion constrain farmers’ behavior. The second reason is that as rational economic agents, farmers consciously avoid such opportunistic behavior when they transfer their land, for example, by transferring the land to relatives rather than to other people with whom they are more distant [25].

From the regression results of the control variables, there is no significant effect of the personal characteristics of the household head on the intensity of fertilizer application by farm households. This is consistent with the findings of Gao [8]. It is also worth noting that the number of plots is also positively related to the intensity of fertilizer application. This may be because the number of plots represents, to some extent, the degree of fineness of the land owned by the farmer. In the case of incomplete land, the application of fertilizer is a more productive means of increasing yields than agricultural machinery [33,34].

### 4.2. Mediating Effect Analysis

This section first examines the mediating effect of land size in the effect of land transfer on fertilizer application intensity. In this paper, farmers’ land size (Landsize) is measured by the area of land operated at the end of the year and introduced into the model as a mediating variable for testing. The test results are shown in Table 5. Column (2) indicates that the level of land transfer has a significant positive effect on the land size of farm households. More important is the result reported in Column (3). This shows that when the variable Landsize is included, the absolute value of the coefficient decreases, although the effect of land transfer level on fertilizer application intensity is still significantly negative. This indicates that land size plays a partial mediating effect. That is, land transfer reduced fertilizer application intensity by increasing the land size of farmers.

These results are consistent with the findings of previous studies. It has been shown that when land size increases, even if the intensity of fertilizer application decreases, it does not negatively affect the output [2]. The main reason for this is that farmers have more room for technology selection when the land size increases. On the one hand, some of the technologies used to replace fertilizers can only be applied on a larger scale [7]; on the other hand, the use of high-level agricultural technologies requires certain fixed capital inputs. As the size of land increases, the cost of this fixed capital will be diluted [8]. In addition, in the context of China, an increase in land size will also bring about a change in the farmer’s identity. This is first manifested in the market, where an increase in the scale of production means an increase in bargaining power. This is when farmers have more incentives to improve the quality of their products and market competitiveness by reducing fertilizer application. The shift in farmer identity also manifests itself in production. Some farmers begin to transform into professional farmers who care more about long-term gains, and their fertilizer application intensity decreases accordingly [37].

We next examine the mediating effect of the mechanization level (Mechan) in land transfer affecting fertilizer application. This paper measured the mechanization level of farm households by the farm machinery operation fee and introduced it as a mediating variable in the analysis. Table 5 reports the regression results. Column (4) shows that land transfer increases the mechanization of the farmer in production. However, Column (5) shows that when both the land transfer level and mechanization are introduced into the equation, the regression coefficient of the latter is positive. This means that mechanization increases the fertilizer application intensity, which has a “suppressing effect” in the effect of land transfer on fertilizer application intensity [40,41].

According to previous studies, land transfer provides conditions for mechanized production in agriculture by increasing the size of the land [30]. However, an increase in the level of agricultural mechanization does not necessarily mean a decrease in the intensity of fertilizer application. According to Zhou and Zhou [31], there is an inverted U-shaped relationship between the level of agricultural mechanization and the intensity of fertilizer application. That is, it is positively correlated with fertilizer application intensity when the level of agricultural mechanization is low, while it is negatively correlated with fertilizer application intensity when the level of agricultural mechanization is high. Therefore, the relationship between agricultural mechanization and fertilizer application is complementary, rather than substitution until the level of agricultural mechanization is developed to a certain extent. This explains our findings to a large extent. Under the household contract system, the level of agricultural mechanization in China has been low due to the fine-grained nature of the land [3]. At this point, the expansion of the production scale brought by land transfer, although potentially increasing the mechanization level of agricultural production, would simultaneously increase the intensity of fertilizer application by farmers.

### 4.3. Heterogeneity Analysis

Food crops and cash crops are very different in terms of various characteristics and cultivation methods. In addition, they each require very different production materials. To examine whether the effect of land transfer on fertilizer application intensity is heterogeneous across crops, we divided the sample according to the type of cultivation reported by farmers in the questionnaire. The regression results for the subsamples are shown in Columns (1) and (2) of Table 6. The effect of land transfer level on fertilizer application intensity is still significantly negative in the food crop subsample, while it is no longer significant in the cash crop subsample.

These results are consistent with previous studies [42]. Possible reasons for these results are as follows: first, since cash crops are relatively more demanding in terms of product quality, this itself inhibits farmers’ fertilizer application behavior [43]; second, the production process of cash crops is more refined, and farmers do not easily change their past production and factor input practices because of the transfer to the land. This is also supported to some extent in Table 6. In contrast to food crops, the intensity of fertilizer application for cash crops is highly correlated with whether the household head has received agricultural training.

The topography and cropping structure vary greatly among different regions of China, and the mode and scale of land transfer occurring in different regions also vary. It is necessary to examine the effects of land transfer levels on fertilizer application in different regions. Specifically, this paper divides the sample according to northeastern, eastern, western, and central regions and performs regressions separately. The regression results are shown in Columns (3)–(6) of Table 6. From the results, the regression coefficients of the eastern region subsample are no longer significant. Among the other three regions, the effect of land transfer on fertilizer application intensity was greatest in the west, followed by the center, and finally the northeast.

The effect of land transfer on the intensity of fertilizer application was not present in eastern China. This is because this region is relatively affluent and has reached a high level of agricultural development. At this time, even with new land transfers, farmers will not easily change their past production habits [42]. Differences in the effects of the land transfer on fertilizer application intensity in the other three regions may stem from the difference in land fragmentation in different regions [43]. According to the mediating effect analysis above, land transfer reduces the intensity of fertilizer application by increasing land size. This means there is much less room for this mechanism to work in the northeast than in the west because the northeastern region of China has vast plains where most of the large farms are located; in the western region, on the other hand, the land fragmentation is quite severe. Therefore, compared to the northeast, land transfer in western China is more likely to result in a certain scale of land fragmentation, thus reducing the intensity of fertilizer application.

## 5. Conclusions

Reducing fertilizer use is critical to reducing rural pollution and ensuring food security. This paper verifies the impact of land transfer on fertilizer application with micro panel data from China. The results show that (1) land transfer significantly reduces fertilizer application intensity; (2) land transfer increases land size and farmers’ use of machinery, but only the increase in land size further reduces fertilizer application intensity; (3) in terms of crop type, the effect of land transfer on fertilizer application intensity is significant only in food crops and not in cash crops; (4) the effect of land transfer on fertilizer application intensity is not significant in the eastern part of China, and the greatest effect is found in the western part.

In a developing country such as China, land reform and promotion of land transfer are primarily aimed at promoting agricultural productivity. However, this study shows that land transfer can reduce fertilizer application and thus rural pollution. Although the policy has yielded unexpected by-products, this not entirely surprising. When the land transfer is suppressed, farmers actually face more potential constraints in running their farms. The best way to increase yields at this point is to use more fertilizer. Moreover, as land transfer proceeds, farmers are given more opportunities to optimize resource allocation and factor inputs, and the excessive fertilizer application in the past is corrected. Therefore, the logic of land transfer to curb fertilizer application is actually the logic of the market economy. Reducing more constraints and thus allowing more productive resources, including land, to flow is important not only for improving production efficiency but also for achieving green development.

Although the analysis of panel data using a fixed-effects model in this paper can largely alleviate the endogeneity problem caused by omitted variables in the estimation, there may still be an inverse causal relationship between land transfer and fertilizer application intensity. Identification of the causal relationship between the two requires further research. Moreover, this paper explores the effects of two mechanisms, land size and mechanization, but in addition to them, the land transfer can also affect fertilizer application intensity through other mechanisms. Analyzing and testing these mechanisms is our future research direction.

## Figures and Tables

**Table 1:** Sample distribution of different land transfer levels.

Year	Total Sample Size	Number of Samples without Transferred Land	Number of Samples with Transferred Land	Sample Size at Different Transfer Levels			
				1	2	3	4
2011	12,697	9270	3427	2746	586	93	2
2012	12,607	9362	3245	2497	641	103	4
2013	12,298	9135	3163	2435	615	112	1
2014	10,646	7837	3809	2087	595	120	7
Total	48,248	35,604	12,644	9765	2437	428	14

**Table 2:** Definition and measurement of control variables.

Variables	Measurement
Age of head of household (Age)	years old
The educational level of head of household (Edu)	years of education
Household income (Income)	ln (total annual household income)
Number of plots (Plotsnum)	piece
Percentage of agricultural labor time (Agrilabor)	household agricultural labor time/household total labor time
Operating land area at the end of the year (Endland)	mu
Fertilizer price (Fprice)	yuan/kg

**Table 3:** Average value of the intensity of variables.

		Different Levels of Land Transfer			
	Total	1	2	3	4
IFA (kilogram/mu)	111.2891	125.3711	67.74267	40.34565	38.08799
Age (years old)	53.75862	54.654	50.88796	49.53735	50.14286
Edu (years)	6.876702	6.868949	6.830148	7.317191	6.769231
Train	0.1522199	0.1332561	0.2139554	0.2330097	0.1428571
Plotsnum (piece)	7.599885	7.093539	9.209711	9.509434	14.85714
Agrilabor (%)	0.5637367	0.5233744	0.6823874	0.8184432	0.7476762
Endland (mu)	17.17683	8.269872	35.16028	109.4857	277.3643

**Table 4:** Level of land transfer and intensity of fertilizer application.

	(1)	(2)	(3)
	IFA	IFA	IFA
RTL	−61.69 ***		
	(15.23)		
RTL1		−28.64 ***	
		(5.833)	
RTL2			−22.56 ***
			(3.521)
Age	−0.0611	−0.102	−0.0645
	(0.474)	(0.371)	(0.473)
Edu	1.768	0.323	1.622
	(1.678)	(1.792)	(1.675)
Train	5.572	9.653	5.215
	(7.626)	(8.600)	(7.611)
Income	37.34 ***	51.38 ***	39.46 ***
	(4.528)	(4.309)	(4.539)
Plotsnum	2.753 ***	−3.490 ***	3.496 ***
	(0.630)	(0.873)	(0.645)
Agrilabor	−21.88 ***	−12.672	−23.62 ***
	(8.456)	(8.207)	(8.445)
Fprice	−0.904 ***	−1.090 ***	−0.913 ***
	(0.205)	(0.298)	(0.204)
Year	control	control	control
Constant	−271.3 ***	−370.9 ***	−297.1 ***
	(56.23)	(51.34)	(56.20)
Obs	10,801	40,127	10,801
R-squared	0.022	0.010	0.026

Notes: This table shows the results of the impact of the level of land transfer on the intensity of fertilizer application using the fixed effects model. Column (1) reports the results using the intensity of fertilizer application as the dependent variable, while Columns (2) and (3) report the results using whether the farmer transferred to the land or not, and the total area of land transferred by the farmer as the dependent variable, respectively. T-statistics are reported in parentheses; *** denotes significance at the 0.01 level. RTL1 is measured by whether the farmer transferred to the land or not (yes = 1; no = 0), and RTL2 is measured by the total area of land transferred by the farmer.

**Table 5:** Mediating Effect Analysis.

	(1)	(2)	(3)	(4)	(5)
	IFA	Landsize	IFA	Mechan	IFA
RTL	−61.69 ***	25.80 ***	−48.85 ***	0.733 ***	−76.18 ***
	(15.23)	(1.009)	(16.04)	(0.0859)	(11.92)
Landsize			−0.498 **		
			(0.195)		
Mechan					3.642 *
					(2.110)
Age	−0.0611	−0.0196	−0.0708	−0.000991	−0.269
	(0.474)	(0.0314)	(0.474)	(0.00283)	(0.390)
Edu	1.768	−0.154	1.692	0.0223 **	0.277
	(1.678)	(0.111)	(1.678)	(0.00971)	(1.336)
Train	5.572	−0.536	5.306	−0.150 ***	7.454
	(7.626)	(0.505)	(7.624)	(0.0422)	(5.812)
Income	37.34 ***	3.796 ***	39.23 ***	0.176 ***	15.05 ***
	(4.528)	(0.300)	(4.586)	(0.0263)	(3.642)
Plotsnum	2.753 ***	0.811 ***	3.157 ***	0.0201 ***	5.072 ***
	(0.630)	(0.0418)	(0.650)	(0.00336)	(0.464)
Agrilabor	−21.88 ***	−2.043 ***	−22.89 ***	−0.0701	−1.485
	(8.456)	(0.560)	(8.462)	(0.0476)	(6.545)
Fprice	−0.904 ***	−0.0359 ***	−0.922 ***	0.00470 *	−2.014 ***
	(0.205)	(0.0136)	(0.205)	(0.00268)	(0.369)
Year	control	control	control	control	control
Constant	−271.3 ***	−38.77 ***	−290.6 ***	4.160 ***	−68.01
	(56.23)	(3.725)	(56.71)	(0.328)	(46.02)
Obs	10,801	10,801	10,801	8080	8080
R-squared	0.022	0.232	0.023	0.109	0.044

Notes: This table shows the results of the mediating effect analysis. T-statistics are reported in parentheses; *** denotes significance at the 0.01 level; and * denotes significance at the 0.10 level. ** denotes significance at the 0.05 level. Farmers’ land size (Landsize) is measured by the area of land operated at the end of the year; the mechanization level (Mechan) is measured by the farm machinery operation fee.

**Table 6:** Heterogeneity analysis.

	(1)	(2)	(3)	(4)	(5)	(6)
	Food Crops	Cash Crops	Northeast	East	West	Central
RTL	−37.04 ***	1.786	−21.57 ***	−142.9	−37.83 **	−25.64 ***
	(10.42)	(36.61)	(8.033)	(100.5)	(18.10)	(9.776)
Age	0.403	0.192	0.192	3.872	−0.00614	−0.428
	(0.327)	(1.150)	(0.278)	(3.071)	(0.499)	(0.326)
Edu	0.907	3.125	4.073 ***	−14.13	5.214 ***	0.784
	(1.213)	(4.263)	(1.208)	(10.07)	(1.596)	(1.371)
Train	−0.621	48.12 **	3.332	7.452	4.154	3.900
	(5.719)	(20.10)	(3.804)	(59.96)	(7.561)	(7.021)
Income	4.645	29.91 ***	6.212 **	169.4 ***	30.31 ***	−3.217
	(3.284)	(11.54)	(2.433)	(25.29)	(5.648)	(3.064)
Plotsnum	1.198 ***	1.387	−0.313	15.29 ***	−2.534 ***	−0.449
	(0.460)	(1.618)	(0.490)	(3.058)	(0.942)	(0.322)
Agrilabor	6.270	−63.92 ***	−3.596	−94.15 *	−16.82 *	−14.55 ***
	(6.042)	(21.23)	(4.137)	(53.31)	(10.15)	(5.597)
Fprice	0.362 **	−2.017 ***	−0.408 ***	−2.520 ***	−2.272 ***	−21.89 ***
	(0.143)	(0.503)	(0.0624)	(0.886)	(0.651)	(1.572)
Year	control	control	control	control	control	control
Constant	49.24	−166.1	−41.08	−1713 ***	−180.6 ***	229.6 ***
	(40.08)	(140.9)	(30.93)	(351.9)	(65.66)	(38.04)
Obs	9587	7587	2564	1620	3794	2679
R-squared	0.020	0.012	0.051	0.100	0.034	0.134

Notes: This table shows the results of the heterogeneity analysis. T-statistics are reported in parentheses; *** denotes significance at the 0.01 level; ** denotes significance at the 0.05 level, and * denotes significance at the 0.10 level.

## Data Availability

The data presented in this study are available on request from the corresponding author. The data are not publicly available due to the database was organized by the Policy Research Office of the Central Committee of the Communist Party of China and the Ministry of Agriculture of China, and are only available to a few research institutions.
